# How Real-Time Case-Based Malaria Surveillance Helps Zanzibar Get a Step Closer to Malaria Elimination: Description of Operational Platform and Resources

**DOI:** 10.9745/GHSP-D-22-00522

**Published:** 2023-10-30

**Authors:** Humphrey R. Mkali, Shabbir M. Lalji, Abdul-wahid Al-mafazy, Joseph J. Joseph, Osia S. Mwaipape, Abdullah S. Ali, Faiza B. Abbas, Mohamed H. Ali, Wahida S. Hassan, Erik J. Reaves, Chonge Kitojo, Naomi Serbantez, Bilali I. Kabula, Ssanyu S. Nyinondi, Mike McKay, Gordon Cressman, Jeremiah M. Ngondi, Richard Reithinger

**Affiliations:** a RTI International, Dar es Salaam, United Republic of Tanzania.; b Zanzibar Malaria Elimination Programme, Ministry of Health, Zanzibar, United Republic of Tanzania.; c U.S. President's Malaria Initiative, U.S. Centers for Disease Control and Prevention, Dar es Salaam, United Republic of Tanzania.; d U.S. President's Malaria Initiative, U.S. Agency for International Development, Dar es Salaam, United Republic of Tanzania.; e RTI International, Research Triangle Park, USA.; f RTI International, Washington, DC, USA.

## Abstract

Testing and treating asymptomatic populations have the potential to reduce the population's parasite reservoir and reduce malaria transmission. Zanzibar's malaria case notification (MCN) platform collects detailed sociodemographic and epidemiological data from all confirmed malaria cases to inform programmatic decision-making. We describe the design and operationalization process of the platform and other malaria surveillance resources that are enabling Zanzibar's progress toward malaria elimination.

The MCN platform consists of an interactive short message service (SMS) system for case notification, a software application for Android mobile devices, a visual question set and workflow manager, a back-end database server, and a web browser-based application for data analytics, configuration, and management. Malaria case data were collected from August 2012 to December 2021 and reported via SMS from all public and private health facilities to a central database and then to district malaria surveillance officers' mobile devices. Data included patient names, shehia (administrative area), and date of diagnosis, enabling officers to track patients, ideally within 24 hours of reporting. Patients' household members were tested for malaria using conventional rapid diagnostic tests (RDTs). Treatment using artemisinin-based combination therapy was provided for persons testing positive.

Between 2012 and 2021, a total of 48,899 index malaria cases were confirmed at health facilities, 22,152 (45.3%) within 24 hours of reporting; 41,886 (85.7%) cases were fully investigated and followed up to the household level. A total of 111,811 additional household members were tested with RDTs, of whom 10,602 (9.5%) were malaria positive.

The MCN platform reports malaria case data in near real time, enabling prompt follow-up of index cases and prompt testing and treatment of members in index case households. Along with routine testing and treatment and other preventive interventions, the MCN platform is foundational to the programmatic efforts in further reducing malaria and ultimately eliminating autochthonous malaria transmission in Zanzibar.

## Abstract (precis)

The authors describe how the Zanzibar Malaria Program successfully implemented a real-time case-based malaria surveillance platform that is helping Zanzibar get closer to malaria elimination.

## Teaser

**Key Findings:**

- Zanzibar's malaria case notification (MCN) platform reports near real-time data on all malaria cases confirmed at public and private health facilities (index cases) and allows for case follow-up and reactive case detection of case household members (secondary cases).
- Continued use of the MCN platform enabled Zanzibar to identify an additional 21.7% malaria cases, with an additional secondary case detected through reactive case detection for every 4.6 index cases.
- Operational challenges affecting the MCN platform's full functionality and operationalization have included shortage of and delays in replacement of hardware, software bugs, strained human resources bandwidth, relevance and use of indicator selection, and data divergence across multiple health information systems.

**Key Implication:**

- To be fully functional and operational, a malaria surveillance platform such as MCN needs adequate resourcing, including timely provision and, as needed, hardware replacement, software maintenance, and adequate number of trained personnel that can manipulate, analyze, use, and act upon the data in the platform, definitions, and inclusion of indicators that are used by malaria programs for evidence-based decision-making and streamlining and interoperability with other health information systems.

## BACKGROUND

Well-implemented surveillance systems are essential for timely programmatic decision-making by national malaria control programs. In malaria elimination settings, the World Health Organization recommends the detection and notification of all malaria cases, their case investigation, the identification of malaria transmission foci, and the application of effective interventions aligned with epidemiological strata.^1^^–^^3^ Active malaria case detection is one of the critical programmatic approaches to achieve malaria elimination. The approach consists of health workers screening at-risk populations for malaria infection regardless of clinical symptoms and treating those testing positive—thereby reducing the parasite reservoir and reducing malaria transmission.^4^^,^^5^ Reactive case detection (rACD) is a type of active case detection whereby all household members and possibly neighbors are screened and treated following the confirmation of an index case in a given household.^4^^–^^6^ Several malaria-eliminating countries in Asia (e.g., Bhutan, Democratic People's Republic of Korea, Indonesia, Malaysia, Philippines, Republic of Korea, Solomon Islands, and Vanuatu)^6^^,^^7^ and Africa (e.g., Eswatini and Zambia)^8^^,^^9^ have been using rACD in their malaria elimination efforts, and—in some cases—all the way to successful elimination (e.g., China and Sri Lanka).^10^^,^^11^

We describe the origins of Zanzibar's malaria surveillance platform and its information technology architecture, highlight other resources for malaria surveillance that were critical to its operationalization, and showcase its functionality by broadly reporting on malaria surveillance trends over the past decade. Studies that have conducted in-depth analyses using the platform's programmatic data are discussed and referenced.

We describe the origins of Zanzibar's malaria surveillance platform and showcase its functionality by reporting on broad malaria surveillance trends over the past decade.

### Setting

The archipelago of Zanzibar is located between longitudes 39.19793 and latitudes 6.16394, 25–50 kilometers off the east coast of the Tanzania mainland in the Indian Ocean. It includes 2 main islands, Pemba and Unguja, with a total land area of 2,461 km^2^ and an estimated population of 1,303,569 people.^12^ Zanzibar comprises 11 districts, which are subdivided into 387 shehias, 258 of which are on Unguja and 129 on Pemba. Shehias are Zanzibar's lowest administrative unit, where many of the public services are planned, managed, and implemented, including for health and malaria. There are 2 main rainfall seasons, the *masika* (March–May) and *vuli* (November–December); rainfall tends to be at its lowest in July. Both rainfall seasons are associated with peak malaria transmission, with the highest malaria case count typically observed in March–May.

## ZANZIBAR MALARIA SURVEILLANCE SYSTEM

After the gradual introduction and scale-up of artemisinin-based combination therapies (ACTs), long-lasting insecticidal nets (LLINs), and indoor residual spraying (IRS) of households with insecticide, malaria prevalence in children aged 5 years and younger decreased from 40% before 2002 to less than 1% in 2007/2008.^13^ This brought Zanzibar to a crossroads, where it could either pursue sustained control or strive for malaria elimination. A malaria elimination feasibility assessment conducted in 2009 indicated that while elimination was possible with the available interventions, pushing for elimination would require a robust surveillance system in addition to strict maintenance of control efforts.^14^

We describe the 3 main elements that together constitute Zanzibar's malaria surveillance system and are critical for its successful operationalization.

### Element #1: The Information, Communication, and Technology Hardware

After the successful scale-up of malaria interventions from 2003 onward,^13^ the Zanzibar Malaria Elimination Program (ZAMEP), formally the Zanzibar Malaria Control Program, developed the Malaria Epidemic Early Detection System (MEEDS) in 2008, where health facility staff used cell phone handsets to submit weekly aggregated case data via short message service (SMS).^15^ After further reduction of malaria prevalence in children aged 5 years and younger to below 1% by 2010, ZAMEP built on the success of MEEDS in 2012 to develop the malaria case notification (MCN) platform (also called Coconut Surveillance, https://coconutsurveillance.org).^16^

**FIGURE 1:**
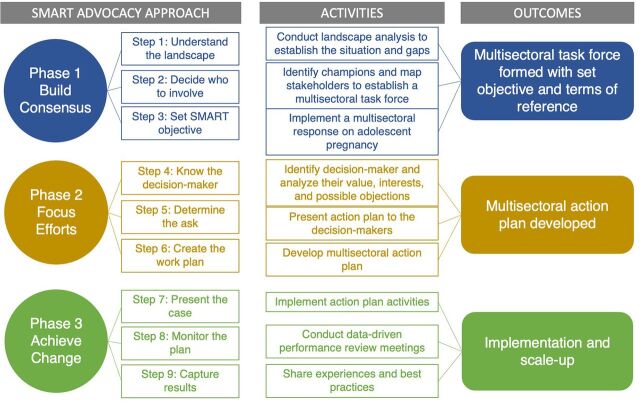
Information Technology Infrastructure of Zanzibar's Malaria Case Notification Platform Abbreviations: rACD, reactive case detection; SMS, short message service; USSD, unstructured supplementary service data.

The MCN platform consists of an interactive SMS system for case notification; a software application for Android mobile devices; a visual question set and workflow manager; a back-end database server; and a web browser-based application for data analytics, configuration, and management (Figure 1). The platform is based on free and open-source software, with the source code repositories publicly available (https://github.com/orgs/Coconut-Data). The source code for the mobile application and web-based analytics application is maintained in the coconut and coconut-analytics repository, respectively; the mobile application plug-in, designed specifically to implement MCN protocols, is maintained in the “coconut-mobile-plugin-zanzibar” repository.

**FIGURE 2:**
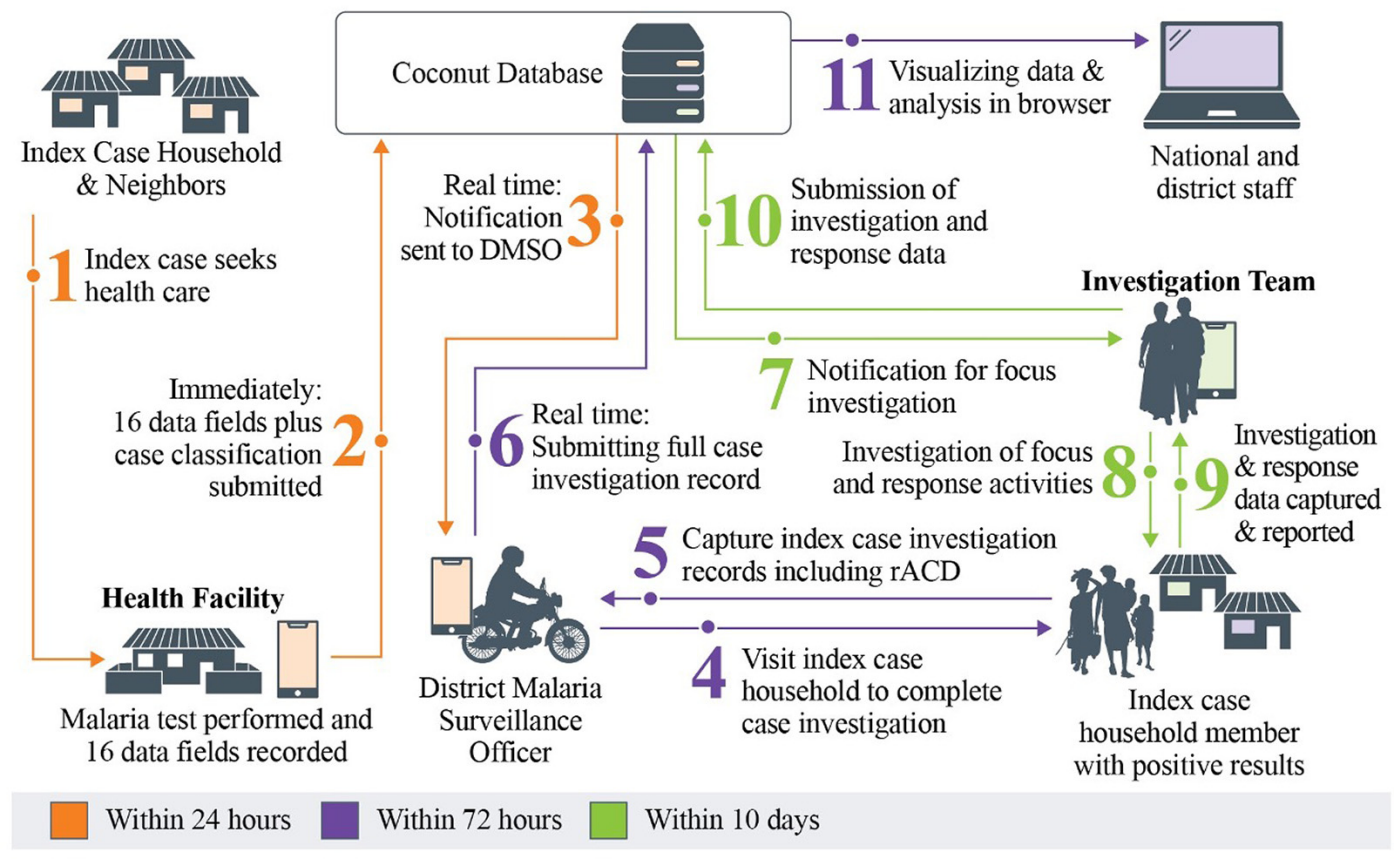
Schematic Framework of Index Case Notification and Household Level Follow-Up and Reactive Case Detection Abbreviations: DMSO, district malaria surveillance officer; rACD, reactive case detection.

The interactive SMS application enables health facility staff to quickly notify the MCN platform of new malaria cases when these are diagnosed by microscopy or rapid diagnostic tests (RDTs) at the health facility level (defined as primary index cases). The mobile application is designed for use by district malaria surveillance officers (DMSOs) as they travel to health care facilities to confirm index cases, collect additional case sociodemographic and epidemiological data, and conduct case follow-up and rACD activities at the household level, including diagnosing secondary cases among the primary index case's household members (Figure 2; Box).

BOX Malaria Case Notification Platform Data Fields and Built-In Reports

**Data Fields**

- **Case Notification**
  - Individual factors: contact information; age; sex; self-reported history of travel in the last 30 days, including destination of travel (country/region); self-reported history of fever in the last 2 weeks; rapid diagnostic test positivity.
- **Case Investigation**
  - Household factors*:* number of people residing in the household; number of household long-lasting insecticidal nets (LLINs); household members' use of LLIN the previous night; household indoor residual spraying application in the last 12 months.
  - Geographical factors*:* household geolocation; weekly rainfall.
- **Focus Investigation**
  - Basic information on the characteristics and location of breeding sites as well as the population of anopheline mosquitoes.

**Built-In Reports:** analysis; foci classification; duration of household investigations; case follow-up status; comparison of weekly facility reports with case follow-ups; epidemic thresholds; morning meeting weekly report; weekly facility report.

The DMSOs use the mobile application to confirm index cases, collect additional case data, and conduct follow-up and rACD activities at household level.

The mobile application is primarily written in CoffeeScript, and both the application and the database server use open-source document-oriented noSQL databases, allowing it to run on commonly available Android mobile devices, including low-cost smartphones and tablets. PouchDB is used to store data on Android mobile devices and to bidirectionally synchronize data between mobile devices and the server's CouchDB database—this enables intermittent online-offline operation and guarantees eventual data consistency. A software plug-in architecture makes it easy to adapt the mobile application to different workflows and users. Thus, a plug-in supports passive case detection (i.e., primary index cases reported by health facilities), rACD at the household level, and other active case detection efforts (e.g., mass/focal screening and treatment); another plug-in is used for entomological investigations, including specimen collection, and managing laboratory results. Mobile device Global Positioning System capability is used to automatically geolocate activities and primary index case households. The advanced bidirectional data synchronization makes it possible to guide field workers based on current response protocols and transmission risk, collaborate on case response, transfer cases, and keep software updated on mobile devices.

A separate software application serves as the visual question set manager. It is used to configure the questions and various workflows used by the MCN platform. It is also used to define the SMS interactions used by health facility staff to report primary index cases. The back-end CouchDB database server manages the central data repository. It can be hosted by a private or public cloud services provider or hosted locally, depending on requirements and resources.

The web browser-based analytics application is written in CoffeeScript and JavaScript to meet the needs of ZAMEP supervisors, providing them with access to data from the mobile application to monitor case response and analyze data in near real time. It includes advanced built-in reports (Box), most of which drill down to case-level detail. It generates routine reports automatically and distributes them to designated users via SMS and email. It also enables users to export case data in CSV file format for analysis in other software programs. This enables supervisors to monitor case response time and completeness as cases progress through the MCN follow-up protocol. Finally, it allows supervisors to analyze the overall efficiency of DMSOs and ZAMEP to help allocate resources effectively.

The mobile application stores data in encrypted form and encrypts data during transmission. Both the mobile and web applications use single-factor role-based security to restrict access to personally identifiable data. The MCN removes all personally identifiable data when generating export data files.

After an extensive pilot phase in 142 facilities and further refinement of the information technology architecture, the MCN platform was progressively scaled up to cover all 189 public and 124 private health facilities on Pemba and Unguja by 2014. In 2019, new features were added to the platform to improve its functionality and data analytics. Features included entomological surveillance and IRS modules, whereby intervention data can be collected, analyzed, and displayed through an interactive dashboard. Other new features included foci and case classification, with the platform automatically classifying cases based on the response of a case's self-reported history of travel in the 30 days before testing positive for malaria. The platform preloads all of the malaria-endemic areas of Tanzania's mainland according to their malaria stratification status (i.e., “high,” “moderate,” “low,” and “very low”)^17^ and classifies whether cases are likely to be imported or locally acquired (autochthonous). Another new feature was a customized weekly report that provides a summary of malaria case yearly trends by week and case classification for Unguja and Pemba. The report provides the output in tables, charts, and maps with the flexibility of navigating to each case for more details.

After an extensive pilot phase, the MCN platform was progressively scaled up to cover all 189 public and 124 private health facilities on Pemba and Unguja by 2014.

Both MEEDS and MCN complement Zanzibar's overarching health management information system, which collects monthly programmatic indicator data from health facilities on a range of diseases and health conditions and has been based on the DHIS2 since 2005. To streamline data systems, steps were taken in 2019 to ensure interoperability of the MCN platform with DHIS2 (see Element #2).

### Element #2: Human and Other Resources

To successfully implement the various malaria surveillance platforms and act on the data they collect and report on, ZAMEP established a new cadre of health personnel in 2012—the DMSOs—supervised by ZAMEP's Surveillance, Monitoring, and Evaluation (SM&E) Unit Team Leader. These DMSOs, of whom there is 1 for each district, are dedicated trained and supervised personnel who: (1) lead district-level SM&E efforts; (2) conduct index case follow-up at the household level; (3) conduct screening of other family members in the index case household; and (4) treat RDT-positive household members with ACTs. Each DMSO is equipped with an Internet-capable Android tablet that runs the MCN surveillance platform, a motorbike, and a backpack containing necessary supplies to conduct malaria rACD (i.e., RDTs, ACTs, personal protective materials such as gloves, etc.). ZAMEP's SM&E Unit convenes all DMSOs bimonthly to review malaria surveillance data, share challenges faced in the field, and orient the DMSOs on any software and other updates of the MCN platform and interactive dashboard; these feedback meetings are complemented by quarterly supervision and mentoring visits of each DMSO by ZAMEP's SM&E Unit. DMSOs undergo a 2-day biannual refresher training, including on any updates to the Zanzibar SM&E guidelines.

The MCN platform has now become an essential tool for ZAMEP in its efforts to eliminate malaria. ZAMEP's SM&E Unit began with 4 staff (3 in Unguja and 1 in Pemba) in 2012, and the number increased to 10 staff (7 in Unguja and 3 in Pemba) as of 2021. Similarly, the number of DMSOs was doubled from 10 to 20 in 2015 and again to 28 in 2019 to provide sufficient personnel to successfully execute Zanzibar's malaria surveillance strategy, particularly in the highest malaria incidence districts (i.e., Central, Urban, West A, and West B).

### Element #3: Strategic and Policy Framework

As a result of the 2009 malaria elimination feasibility assessment, Zanzibar changed the name of the Zanzibar Malaria Control Program to ZAMEP in 2013. In 2015, the World Health Organization conducted a malaria elimination audit in Zanzibar^18^ and recommended the establishment of a high-level elimination advisory committee, the Zanzibar Malaria Elimination Advisory Committee (ZMEAC)—an independent group of local and international malaria experts who meet biannually and provide technical guidance to ZAMEP on implementation of malaria elimination interventions and continuously review Zanzibar's progress toward elimination. ZAMEP convened the first ZMEAC meeting in August 2018. In July 2021, ZAMEP revived the malaria surveillance, monitoring, and evaluation technical working group (SME-TWG)—a group of local malaria stakeholders that provides strategic advice and technical inputs on a quarterly basis and supports ZAMEP to follow up and act on ZMEAC's feedback and recommendations.

Zanzibar's approaches to achieve its malaria elimination goal are anchored in the 2013–2018 National Malaria Strategic Plan,^19^ updated and renewed in 2017 for the 2018–2023 period.^20^ In addition, the national strategic plan is complemented by the following normative guidelines and documents: Malaria Diagnosis and Treatment Guidelines (2018); Zanzibar Malaria Elimination Communication Strategy (2018–2023); Foci Entomological Investigation and Response Standard Operating Procedures (2020); Guidelines for Larval Source Management (2020); Guidelines for Vector Control in Malaria Elimination (2017); Insecticide Resistance Mitigation Plan (2016); National Guidelines for Malaria Surveillance and Response in Zanzibar (2016); Malaria Surveillance in Zanzibar Field Manual for Health Facilities, District Malaria Surveillance Officers and Surveillance Monitoring and Evaluation Team (2016); Guidelines for District Malaria Response Team (2016); and Standard Operating Procedures for Data Analysis and Interpretation (2016).

## HOW IT ALL COMES TOGETHER

Figure 2 shows the schematic framework of index case notification and follow-up at the household level. All confirmed malaria cases on Zanzibar are captured by the MCN platform. Suspected malaria cases access health facilities, where they get tested for malaria by either microscopy or RDT. If confirmed as positive, the health provider prescribes ACTs per National Malaria Diagnosis and Treatment Guidelines. Within 24 hours of the index case being detected at the facility level, the provider sends an unstructured supplementary service data notification to a central ZAMEP computing server. The DMSO receives the forwarded notification on their mobile phone and Android tablet and, within 24 hours, visits the health facility to confirm the reported index case and collect additional information, including the patient's contact details. Within 72 hours of being notified, the DMSO follows up index cases at the household level, ensuring the patient is adhering to prescribed treatment and investigating case details that will inform case classification (e.g., whether the case was autochthonous versus possibly imported based on travel history in the preceding 30 days). DMSOs then use RDTs to screen all the additional household members; members with a positive RDT result (i.e., secondary cases) are treated with an ACT. Through a standardized electronic questionnaire completed by the DMSO on their Android tablet, the case follow-up and rACD data, including for household-based screening and treatment, are linked to each index case via the MCN platform. Specific variables collected in the questionnaire are individual, household, and geographical factors (Box).

The data collected by DMSO are then uploaded to the ZAMEP server, where they can be accessed by the ZAMEP SM&E Unit—including through automated and standardized tables, charts, and maps—for focused review and programmatic decision-making.

## AT SCALE: 10 YEARS OF IMPLEMENTATION

### Malaria Case Notification and Household Follow-Up: Index Cases

**FIGURE 3:**
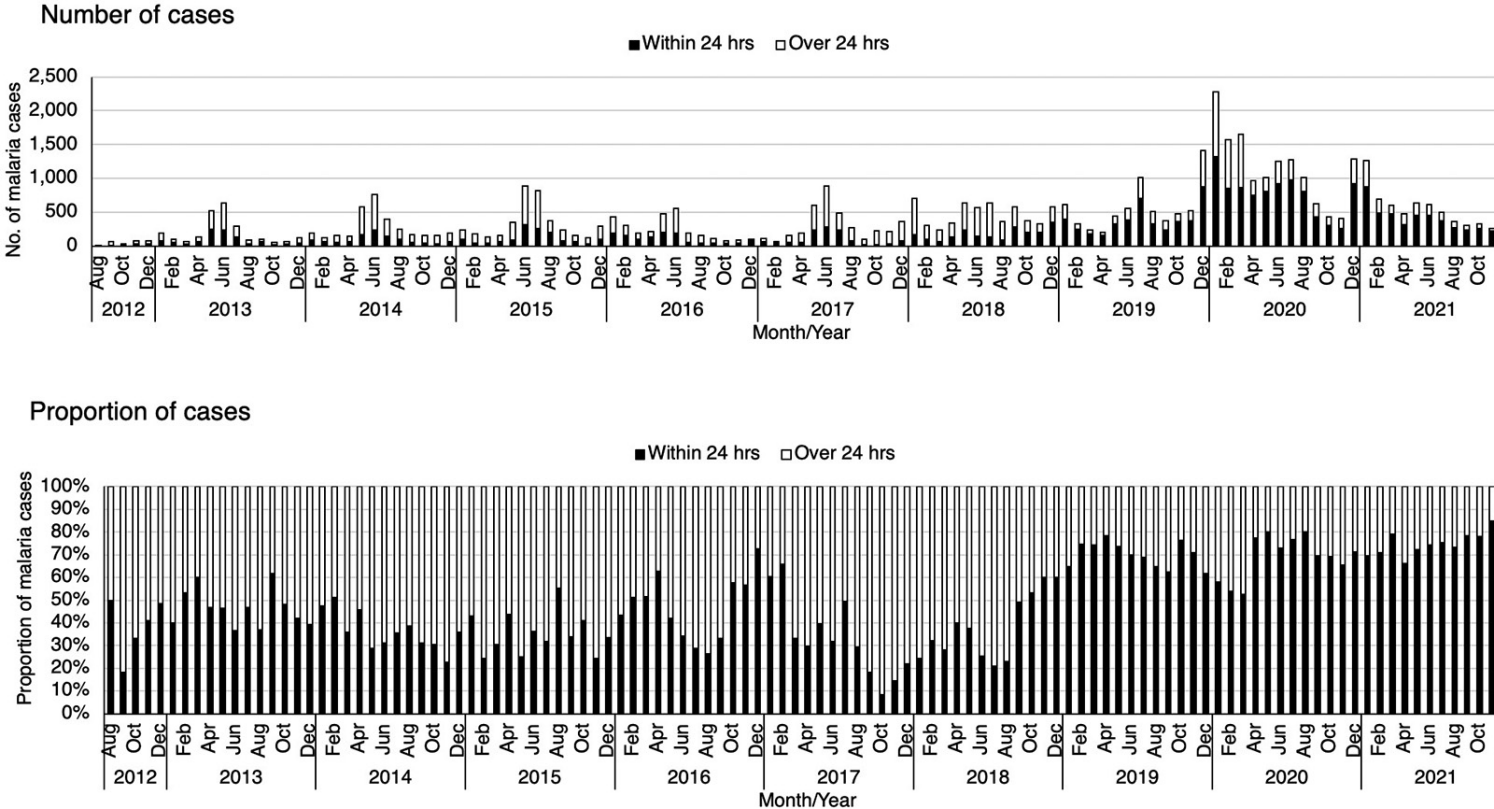
Number and Proportion of Malaria Index Cases Notified Within 24 Hours of Diagnosis, 2012–2021, Zanzibar

Between 2012 and 2021, DMSOs were notified that a total of 48,899 index cases were confirmed at public and private health facilities, 22,152 (45.3%) within 24 hours of reporting (Figure 3). Median age of index cases was 21 (interquartile range: 12–30) years, and 25,418 (51.9%) were male. Of index cases, 41,886 (85.7%) were followed up to the household level; the proportion of followed-up index cases was moderately correlated with the number of district-level index cases notified (Spearman *rho*=0.52). Household investigations were completed for 38,965 (79.7%) index cases that had been followed up. Annual peaks in index case notifications generally coincided with Zanzibar's 2 transmission periods (January–March; May–August), which follow the 2 main rainfall seasons (vuli, November–January; masika, March–May).

### Key MCN Platform Performance Metrics: Case Notification, Case Investigation, and Household Investigation

The time from the initial date of malaria diagnosis of an index case at the health facility level to the time when the case was reported to the MCN platform (target: within 24 hours/1 day) represents the timeliness of case notification. Between 2012 and 2021, health providers notified DMSOs of 22,152 (45.3%) index cases within 24 hours of being diagnosed at the health facility level. Timeliness of index case notification improved over time, with the proportion of cases notified within 24 hours increasing from 36.1% (90/249) in 2012 to 72.7% (4,679/6,436) in 2021 (Figure 3).

Timeliness of index case notification improved over time, with the proportion of cases notified within 24 hours increasing from 36.1% in 2012 to 72.7% in 2021.

**FIGURE 4:**
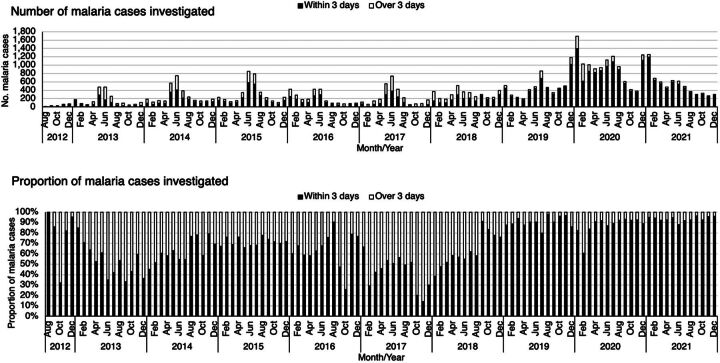
Number and Proportion of Malaria Index Cases Investigated After Notification, 2012–2021, Zanzibar

The time from case notification to when the household visit of the index cases was completed (target: within 72 hours/3 days) represents the timeliness of case investigation. Between 2012 and 2021, the proportion of malaria index cases followed up to the household level within 72 hours of diagnosis was 32,097 (76.6%), increasing from 79.5% (144/181) in 2012 to 91.8% (5,909/6,436) in 2021 (Figure 4).

**FIGURE 5:**
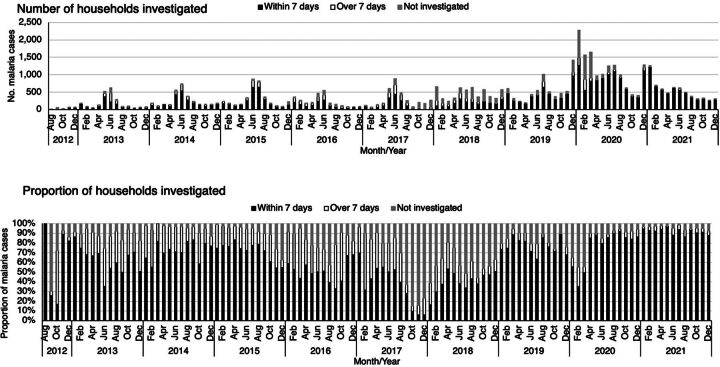
Number and Proportion of Malaria Index Case Households Investigated After Notification, 2012–2021, Zanzibar

The time from case notification to the focus investigation of index case household members (target: within 168 hours/7 days) represents the timeliness of household investigation. Of the 38,965 index case household investigations that were completed over the entire study period, 32,257 (65.9%) were done within 7 days, increasing from 59.8% (149/249) in 2012 to 92.4% (5,945/6,436) in 2021 (Figure 5).

### Malaria Positivity Among Index Case Household Members: Secondary Cases

**FIGURE 6:**
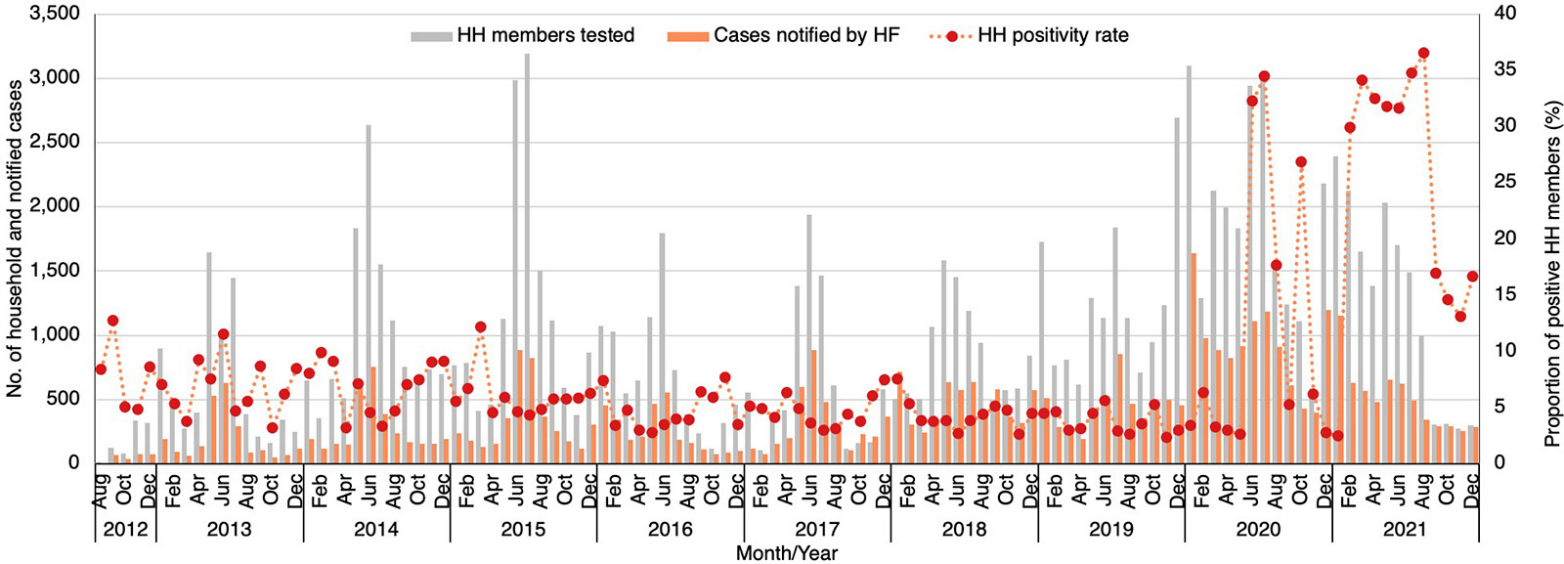
Number of Malaria Cases Notified, Household Members Tested, and Positivity Rate, 2012–2021, Zanzibar Abbreviations: HF, health facility; HH, household.

From 2012 to December 2021, DMSOs tested a total of 111,811 household members of index cases for malaria using RDTs, of whom 10,602 (9.5%) were RDT positive. Test positivity rate varied by district, ranging from 4% in Micheweni district to 14.3% in Kusini district. Across study years, the test positivity rate among household members ranged from 2.3% in November 2019 to 36.5% in August 2021 (Figure 6); the resurgence of malaria since 2019 is likely associated with increased rainfall in both masika and vuli rainfall seasons.^21^ Overall, 21.7% additional malaria cases were identified through rACD, ranging from 12.7% in Micheweni to 27.8% in Kusini. Conversely, this means that for every 4.6 index cases, an additional secondary case was detected through household-level rACD using a conventional RDT.

### History of Travel Among Index Malaria Cases

**FIGURE 7:**
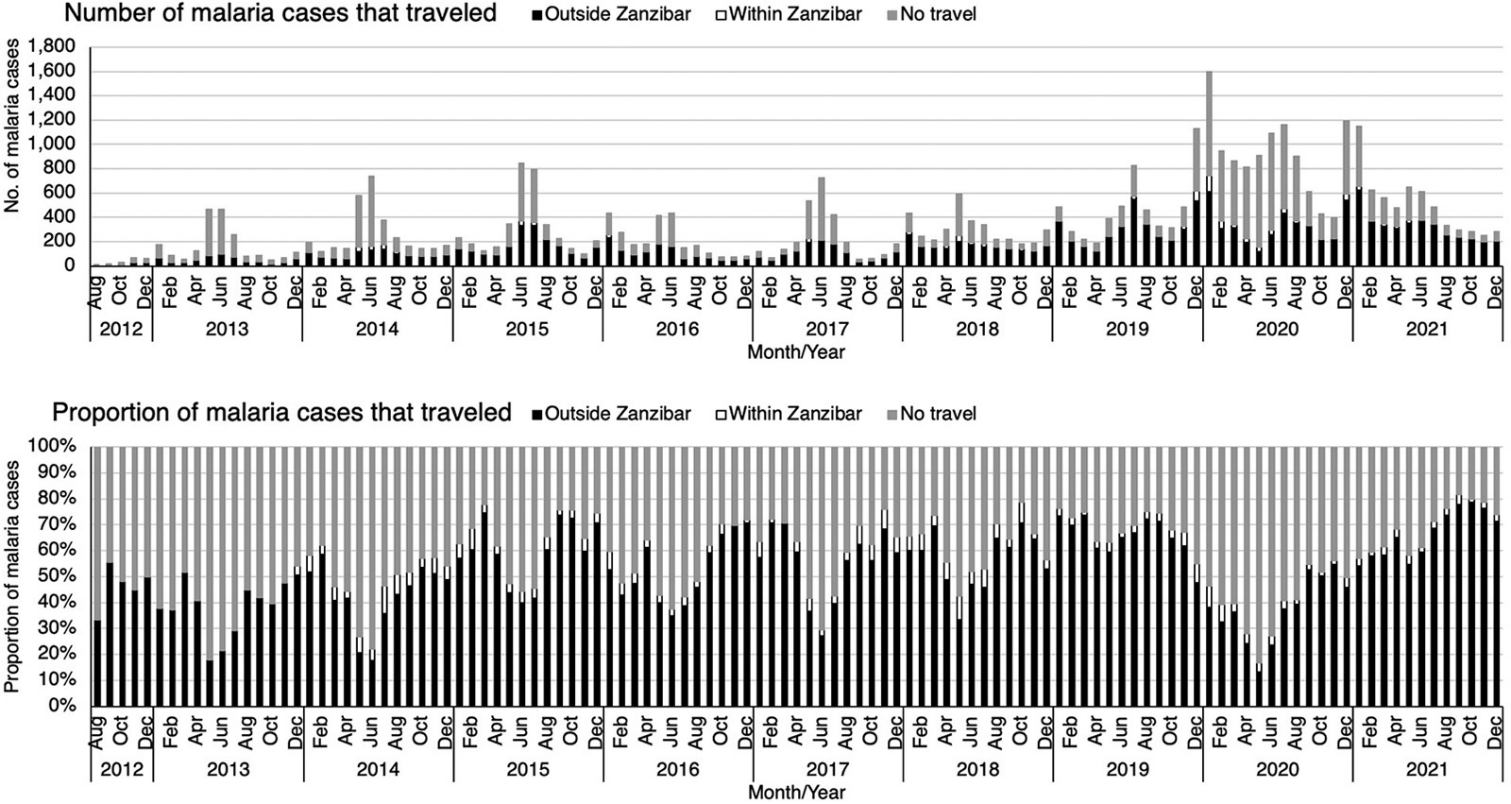
Number and Proportion of Malaria Cases With Self-Reported Travel History in 30 Days Before Diagnosis, 2012–2021, Zanzibar

History of travel is defined as self-reported travel outside or within Zanzibar in the last 30 days before testing for malaria. Of the 40,456 index malaria cases for which self-reported travel history data were available, 19,235 (47.5%) reported having traveled outside of Zanzibar in the 30 days before diagnosis. The proportion of malaria cases with a self-reported history of travel varied throughout the years; it was lowest (30.4%) in 2013 and highest (63.2%) in 2019 (Figure 7).

### LLIN Use and IRS Coverage Among Index and Secondary Malaria Cases

**FIGURE 8:**
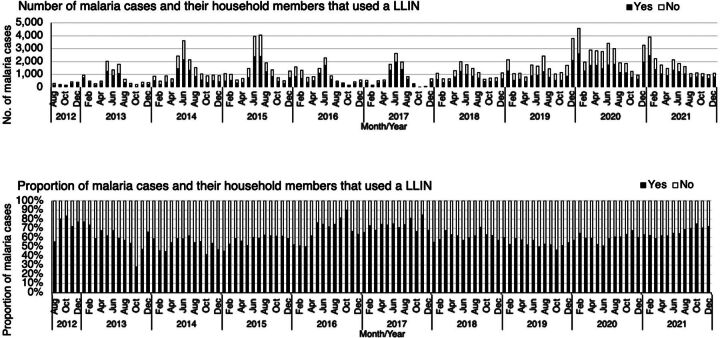
Number and Proportion of Malaria Cases and Their Household Members With Self-Reported LLIN Use, 2012–2021, Zanzibar Abbreviation: LLIN, long-lasting insecticidal net.

LLIN use is defined as self-report of having slept under an LLIN the previous night; coverage of IRS in households is defined as spraying of residual insecticides within the last 12 months. Of 153,697 investigated index cases and tested household members for which self-reported LLIN use data were available, 90,929 (59.2%) reported using an LLIN the night before diagnosis. LLIN use was lowest in 2019 (54.5%) and highest in 2012 (75.6%) (Figure 8). Of the 34,637 index malaria case households investigated, 19,959 (57.6%) reported having their household sprayed with residual insecticide in the last 12 months; the proportion of households covered by IRS was lowest in 2020 (48.8%) and highest in 2018 (85.5%).

### The Added Value of the MCN Platform

Although the primary use of the MCN platform is to implement timely and effective rACD, it also allows for enhanced malaria risk stratification, more effective targeting of existing and new malaria interventions, as well as monitoring the programmatic coverage of existing interventions—an added value that ZAMEP fully recognized in the 2018–2023 NMSP by emphasizing the crucial role that malaria surveillance plays in its efforts to eliminate malaria from the archipelago. Thus, the MCN platform data were used by ZAMEP to reduce IRS coverage from district to shehia and then community level (i.e., so-called “reactive focal IRS”). Similarly, drops in LLIN use triggered community sensitization efforts to increase use to greater levels or determine whether focal distribution of new LLINs was required because existing LLINs were worn. After a more granular risk stratification, focal mass drug administration will be implemented as an additional malaria intervention starting in early 2024.

Similarly, because each followed-up index case and secondary case is geotagged in space and time, along with their socioeconomic and epidemiologic characteristics, the MCN platform allows for advanced analytics to assess risk factors of infection,^22^ determine spatiotemporal transmission dynamics and identify malaria hotspots,^21^ or model different intervention scenarios.^23^

rACD in Zanzibar is relatively inexpensive as a surveillance response system, costing about US$0.16 per person.^24^ The cost per detected case (∼US$570) is comparable with that reported from other rACD systems.^25^^,^^26^ Overall, during the 2012–2021 period, an additional 21.7% malaria cases were identified through rACD, which corresponds to an additional secondary case being detected through household-level rACD for every 4.6 index cases.

## IMPLEMENTATION CHALLENGES AND LESSONS LEARNED

Engagement between ZAMEP and stakeholders across all administrative and programmatic levels when developing, testing, and deploying the MCN platform has been critical to effectively cultivate ownership, as well as gradually ensure the transfer of skills and knowledge at all levels necessary to maintain and operate the MCN platform. The platform's successful implementation over the past 10 years has arguably supported Zanzibar to better characterize its malaria epidemiology—that is, the distribution of cases in space and time, as well as allow for more timely detection of case upsurges and a more effective malaria response. The main implementation challenges and lessons learned following the full operationalization of the MCN platform can be grouped into 6 major categories.

The MCN platform's successful implementation over the past 10 years has arguably supported Zanzibar to better characterize its malaria epidemiology.

### Shortage of and Delays in Replacement of Malfunctioning Hardware

Any new surveillance platform requires not only infrastructure (e.g., smartphones or tablets and chargers) but also mobile data capacity and connectivity to effectively leverage the benefits of mobile technology. Gaps identified through routine monitoring over the years include delayed replacement of damaged or lost smartphones, tablets, and chargers; delayed distribution of mobile cards; and cell service blackouts—all of which affected timely and complete reporting of cases at health-facility level or follow-up of index cases and investigations by DMSOs to household level. Additionally, DMSOs' follow-up of cases was affected by nonfunctional motorbikes used for transportation and fuel shortages.

### Software Maintenance

Similarly, any software-based platform requires constant maintenance to run smoothly on a day-to-day basis, and a remote helpdesk was available to ZAMEP whenever a software issue was detected and reported. Thus, for example, from late October to early November 2017, the platform experienced several bugs due to the incompatibility of the existing software with Chrome and Android security upgrades, which were essential for capturing geolocational information of cases and households.

### Strained Human Resources Bandwidth

Timeliness and completeness of case and household investigation are important performance measures of any disease surveillance system.^4^^,^^14^ Although we show that the MCN platform's performance measures steadily improved since 2012 (i.e., it enabled ZAMEP to detect an additional 10,602 cases among followed-up household members), we also show that timely index case notification and follow-up was a challenge, particularly during peak transmission season when health facilities and DMSOs were struggling to respond to and follow up the high volume of index cases attending or being notified by health facilities. To minimize non-follow-up of index cases, ZAMEP regularly made health service providers aware of the importance of accurate recordkeeping (e.g., during case management trainings and supportive supervision visits), as well as tried to reduce DMSO bandwidth constraints (e.g., by increasing the number of DMSOs from 10 in 2012 to 28 in 2019). Additionally, new features to the MCN platform now negate the need for DMSOs to return to health facilities to confirm cases and collect additional case information; rather, all data fields are electronically sent to the DMSOs, and from there, they can directly visit an index case's household for case and focus investigation—saving time and resources.

### Diagnostic Limitations for Identifying Secondary Cases

Conventional RDTs are currently being used to confirm secondary cases (in most cases and years, the Standard Diagnostics Bioline HRP2/pLDH RDT was used). Several studies^27^^–^^29^ have shown the limitations of the HRP2/pLDH RDTs in diagnosing these cases, who are often asymptomatic and have low parasitemia. Certainly, a more sensitive diagnostic test would increase the yield and lower the cost of each additional secondary case detected and, thus, the rACD approach. Unfortunately, a more sensitive, quality-controlled, easy-to-use, and low-cost diagnostic alternative that could be used at scale is not available. Given that secondary cases tend to cluster within or in the vicinity of an index case's household, discussions have been ongoing to assess reactive MDA as an intervention that would cover these households and, thereby, prevent development of malaria symptoms and possible parasite transmission if exposed to anopheline vectors.

### Inclusion of Proxy Indicators That Are Sensitive yet Not Specific

As previously reported,^21^^–^^23^ analysis of the MCN data suggests that travel is a major driver of malaria cases in both high and low transmission periods. Yet, we cannot state that these cases are truly imported due to the long time period covered by the self-reporting (i.e., 30 days before being diagnosed with malaria) and the possibility of these cases having been infected locally. To ascertain whether cases with a travel history are truly imported, more advanced approaches such as whole-of-genome sequencing^30^ would need to be used routinely, which is not feasible on a large scale due to infrastructure requirements and costs. Nonetheless, because of the association of malaria with travel, targeting key traveler groups to limit malaria importation is being discussed by ZAMEP with stakeholders, including education about disease risk and implementation of protective measures (e.g., use of mosquito repellents, LLINs, or chemoprophylaxis) as well as malaria screening and treatment at major ports of entry (e.g., airport and main ferry terminals).

### Proliferation of Health Information Systems and Data Divergence

In Zanzibar, other health program and malaria surveillance data have traditionally been captured in separate systems. Since 2005, Zanzibar's Ministry of Health has used DHIS2 as its main tool for data collection and aggregation and its national repository for health data. Meanwhile, both malaria surveillance systems (MEEDS and MCN) are independently run by ZAMEP, and before their integration, these systems did not share data with each other, making it more difficult to combine data from both sources for effective health program management and evidence-based decision support. Because these 3 platforms use separate information technology architecture and report on different time windows (i.e., monthly for DHIS2, weekly for MEEDS, and daily or real-time for MCN), data divergence occurred. In 2019, the Ministry of Health recognized the value of bringing the data from these systems together and making the platforms interoperable. Working in collaboration with several partners and stakeholders, the Ministry of Health decided to integrate the DHIS2 and MCN platforms. The overall goal of this effort was to facilitate evidence-based decision-making by health teams down to the district, shehia, and facility levels by providing access to combined data from both systems presented in user-friendly dashboards built around selected key indicators. Once system interoperability was achieved, data from MCN could be sent to DHIS2 via a web application programming interface. As a result, DHIS2 became the final destination for both malaria surveillance and other health data for analysis and program management. Integration was completed in April 2020, when Zanzibar's system was successfully able to push data from MCN to DHIS2.

After the continued successful implementation of MCN, ZAMEP set performance standards for malaria index case notification and follow-up. For case notification, the target is to notify 90% of cases within 24 hours of diagnosis at the health facility level. For case follow-up, the target is to follow up 100% of notified cases, with 90% of cases to be followed up within 48 hours of notification.^24^ Although timeliness of notification and follow-up have improved considerably over time, more resources will be required to enable ZAMEP to achieve and maintain these current performance standards.

Although timeliness of notification and follow-up have improved considerably over time, more resources will be required to enable ZAMEP to achieve and maintain these current performance standards.

## CONCLUSION

Zanzibar established a fully operational case-based malaria surveillance system in 2012—a critical element in the progress toward malaria elimination. The MCN platform has been successful in enabling not only near real-time reporting of index cases identified at health facilities but also identification and treatment of secondary cases. The data generated through index case and household investigations has facilitated classification of which cases were likely imported (i.e., associated with travel) rather than local (i.e., autochthonous); the monitoring of case trends in specific administrative units; and, more generally, the graphical visualization of all malaria cases in space and time. Data available in the platform have also been used to target and monitor the coverage of vector control interventions, such as LLINs and IRS, as well as enabled ZAMEP to detect and respond to case increases and outbreaks.

Additionally, the data generated by and reported through the MCN platform allow for advanced epidemiological analyses, informing ZAMEP on what type of interventions to implement, when, and where. For example, given the increasing association of cases with travel outside of Zanzibar, data from the platform are now helping ZAMEP to—in collaboration with various stakeholders—develop various approaches to mitigate the impact of travel on malaria case burden (and likely autochthonous transmission). Ultimately, the MCN platform is foundational to all programmatic efforts to further reduce malaria on Zanzibar and ultimately eliminate autochthonous malaria transmission.

## Notes

**Peer Reviewed**

**First published online:** October 20, 2023.

## References

[1] World Health Organization (WHO). *A Framework for Malaria Elimination*. WHO; 2017. Accessed September 28, 2023. https://apps.who.int/iris/handle/10665/254761

[2] World Health Organization (WHO). *Malaria Surveillance, Monitoring and Evaluation: A Reference Manual*. WHO; 2018. Accessed September 28, 2023. https://apps.who.int/iris/handle/10665/272284

[3] World Health Organization (WHO). *Global Technical Strategy for Malaria 2016–2030*. 2021 update. WHO; 2021. Accessed September 28, 2023. https://apps.who.int/iris/handle/10665/342995

[4] World Health Organization (WHO). *Disease Surveillance for Malaria Elimination: An Operational Manual*. WHO; 2012. Accessed September 28, 2023. https://iris.who.int/handle/10665/44852

[5] Sturrock HJW, Hsiang MS, Cohen JM, et al. Targeting asymptomatic malaria infections: active surveillance in control and elimination. PLoS Med. 2013;10(6):e1001467. 10.1371/journal.pmed.1001467. 23853551 PMC3708701

[6] Moonen B, Cohen JM, Snow RW, et al. Operational strategies to achieve and maintain malaria elimination. Lancet. 2010;376(9752):1592–1603. 10.1016/S0140-6736(10)61269-X. 21035841 PMC3037542

[7] Smith Gueye C, Sanders KC, Galappaththy GNL, et al. Active case detection for malaria elimination: a survey among Asia Pacific countries. Malar J. 2013;12(1):358. 10.1186/1475-2875-12-358. 24103345 PMC3852840

[8] Sturrock HJW, Novotny JM, Kunene S, et al. Reactive case detection for malaria elimination: real-life experience from an ongoing program in Swaziland. PLoS One. 2013;8(5):e63830. 10.1371/journal.pone.0063830. 23700437 PMC3658965

[9] Cao J, Sturrock HJ, Cotter C, et al. Communicating and monitoring surveillance and response activities for malaria elimination: China’s “1-3-7” strategy. PLoS Med. 2014;11(5):e1001642. 10.1371/journal.pmed.1001642. 24824170 PMC4019513

[10] Feng X, Huang F, Yin J, Wang R, Xia Z. Key takeaways from China’s success in eliminating malaria: leveraging existing evidence for a malaria-free world. BMJ Glob Health. 2022;7(4):e008351. 10.1136/bmjgh-2021-008351. 35487673 PMC9058700

[11] Perera R, Caldera A, Wickremasinghe AR. Reactive Case Detection (RACD) and foci investigation strategies in malaria control and elimination: a review. Malar J. 2020;19(1):401. 10.1186/s12936-020-03478-0. 33172462 PMC7653886

[12] Tanzania. National Bureau of Statistics (NBS); Office of Chief Government Statistician (OCGS). 2012 Population and Housing Census. NBS/OCGS; 2013. Accessed September 28, 2023. https://catalog.ihsn.org//catalog/4618/download/58601

[13] Björkman A, Shakely D, Ali AS, et al. From high to low malaria transmission in Zanzibar—challenges and opportunities to achieve elimination. BMC Med. 2019;17(1):14. 10.1186/s12916-018-1243-z. 30665398 PMC6341737

[14] Zanzibar Malaria Control Programme. *Malaria Elimination in Zanzibar - A Feasibility Assessment*. Zanzibar Malaria Control Programme; 2009.

[15] RTI International. *Evaluation Report - Malaria Early Epidemic Detection System 2008 - 2013*. RTI International; 2013.

[16] University of California San Francisco. *Surveillance Systems to Facilitate Malaria Elimination*. Global Health Sciences, University of California; 2014.

[17] Thawer SG, Chacky F, Runge M, et al. Sub-national stratification of malaria risk in mainland Tanzania: a simplified assembly of survey and routine data. Malar J. 2020;19(1):177. 10.1186/s12936-020-03250-4. 32384923 PMC7206674

[18] World Health Organization (WHO). *WHO Mission Report of the Zanzibar Malaria Elimination Audit*. WHO/AFRO/IST/ESA/WCO-Tanzania; 2015.

[19] Zanzibar. Ministry of Health (MOH). *Zanzibar Malaria Elimination Strategic Plan III 2013/14-2017/18*. MOH; 2013.

[20] Zanzibar. Ministry of Health (MOH). *Zanzibar Malaria Elimination Strategic Plan IV 2018/19-2022/23*. MOH; 2017.

[21] Bisanzio D, Lalji S, Abbas FB, et al. Spatiotemporal dynamics of malaria in Zanzibar, 2015–2020. BMJ Glob Health. 2023;8(1):e009566. 10.1136/bmjgh-2022-009566. 36639160 PMC9843203

[22] Mkali HR, Reaves EJ, Lalji SM, et al. Risk factors associated with malaria infection identified through reactive case detection in Zanzibar, 2012–2019. Malar J. 2021;20(1):485. 10.1186/s12936-021-04025-1. 34952596 PMC8710018

[23] Das AM, Hetzel MW, Yukich JO, et al. The impact of reactive case detection on malaria transmission in Zanzibar in the presence of human mobility. Epidemics. 2022;41:100639. 10.1016/j.epidem.2022.100639. 36343496 PMC9758615

[24] MEASURE Evaluation. *Operational Research to Increase the Effectiveness of the Malaria Surveillance and Response System in Zanzibar: Final Report*. MEASURE Evaluation; 2018.

[25] Zelman BW, Baral R, Zarlinda I, et al. Costs and cost-effectiveness of malaria reactive case detection using loop-mediated isothermal amplification compared to microscopy in the low transmission setting of Aceh Province, Indonesia. Malar J. 2018;17(1):220. 10.1186/s12936-018-2361-y. 29859081 PMC5984760

[26] Ntuku H, Smith-Gueye C, Scott V, et al. Cost and cost effectiveness of reactive case detection (RACD), reactive focal mass drug administration (rfMDA) and reactive focal vector control (RAVC) to reduce malaria in the low endemic setting of Namibia: an analysis alongside a 2×2 factorial design cluster randomised controlled trial. BMJ Open. 2022;12(6):e049050. 10.1136/bmjopen-2021-049050. 35738650 PMC9226870

[27] Cook J, Aydin-Schmidt B, González IJ, et al. Loop-mediated isothermal amplification (LAMP) for point-of-care detection of asymptomatic low-density malaria parasite carriers in Zanzibar. Malar J. 2015;14(1):43. 10.1186/s12936-015-0573-y. 25627037 PMC4318361

[28] Björkman A, Cook J, Sturrock H, et al. Spatial distribution of falciparum malaria infections in Zanzibar: implications for focal drug administration strategies targeting asymptomatic parasite carriers. Clin Infect Dis. 2017;64(9):1236–1243. 10.1093/cid/cix136. 28431115 PMC5399945

[29] Grossenbacher B, Holzschuh A, Hofmann NE, et al. Molecular methods for tracking residual Plasmodium falciparum transmission in a close-to-elimination setting in Zanzibar. Malar J. 2020;19(1):50. 10.1186/s12936-020-3127-x. 31996210 PMC6988349

[30] Morgan AP, Brazeau NF, Ngasala B, et al. Falciparum malaria from coastal Tanzania and Zanzibar remains highly connected despite effective control efforts on the archipelago. Malar J. 2020;19(1):47. 10.1186/s12936-020-3137-8. 31992305 PMC6988337

